# Efficacy and safety of novel carbapenem–β-lactamase inhibitor combinations: Results from phase II and III trials

**DOI:** 10.3389/fcimb.2022.925662

**Published:** 2022-09-23

**Authors:** Wei Yu, Ping Shen, Qixia Luo, Luying Xiong, Yonghong Xiao

**Affiliations:** State Key Laboratory for Diagnosis and Treatment of Infectious Diseases, National Clinical Research Center for Infectious Diseases, Collaborative Innovation Center for Diagnosis and Treatment of Infectious Diseases, The First Affiliated Hospital, Zhejiang University School of Medicine, Hangzhou, China

**Keywords:** meropenem–vaborbactam, imipenem–cilastatin/relebactam, clinical cure, mortality, adverse effects

## Abstract

**Objectives:**

The addition of novel β-lactamase inhibitors to carbapenems restores the activity against multidrug-resistant Gram-negative bacteria. The aim of this study was to summarize the evidence on the efficacy and safety of novel carbapenem–β-lactamase inhibitor combinations.

**Methods:**

We conducted a meta-analysis of clinical trials comparing novel carbapenem–β-lactamase inhibitor combinations with comparators to assess the clinical and microbiological responses, mortality, and adverse events (AEs).

**Results:**

A total of 1,984 patients were included. The pooled risk ratios (RRs) of clinical cure, microbiological eradication, all-cause mortality, and 28-day mortality were 1.11 (95% CI: 0.98–1.26), 0.98 (95% CI: 0.82–1.16), 0.90 (95% CI: 0.49–0.94), and 0.68 (95% CI: 0.49–0.94) between the novel carbapenem–β-lactamase inhibitor combinations and control groups. Sensitivity analysis revealed that the phase II trial of imipenem–cilastatin/relebactam (ICR) against complicated urinary tract infections could be the most important factor of heterogeneity for the microbiological response. The therapeutic effect of novel carbapenem–β-lactamase inhibitor combinations was better in meropenem–vaborbactam (MEV), phase III trials, and number of patients less than 200. The RRs of AEs from any cause and serious adverse events (SAEs) for patients receiving novel carbapenem–β-lactamase inhibitor combinations were 0.98 (95% CI: 0.93–1.04) and 1.01 (95% CI: 0.75–1.36), respectively.

**Conclusions:**

ICR and MEV were superior to comparators for clinical cure and survival rate in the treatment of complicated infections, and both were as tolerable as the comparators.

## Introduction

Carbapenems remain the most commonly used antibiotic against multidrug-resistant (MDR) infections (El-Gamal et al., 2017). However, the resistance to carbapenems is increasing throughout the world, posing a major public health threat (Papp-Wallace et al., 2011). Fortunately, several novel β-lactam/β-lactamase inhibitors, including ceftazidime–avibactam, ceftolozane–tazobactam, meropenem–vaborbactam (MEV), and imipenem–cilastatin/relebactam (ICR), have been developed to battle resistance against carbapenemase-producing bacteria (Papp-Wallace, 2019). Previous studies have demonstrated that the addition of novel β-lactamase inhibitors significantly improved the activity of carbapenems against *Enterobacteriaceae*-producing extended-spectrum beta-lactamases (ESBLs), AmpC, and *Klebsiella pneumoniae* carbapenemase (KPC) (Castanheira et al., 2016; Carpenter et al., 2019). The approved carbapenem–β-lactamase inhibitor combinations by the Food and Drug Administration (FDA) are MEV and ICR. Vaborbactam is a novel cyclic boronic acid-based β-lactamase inhibitor, while relebactam is a bridged diazabicyclo[3.2.1]octanone non-β-lactam inhibitor (Bush and Bradford, 2019). Both inhibitors display activity against Ambler class A and class C β-lactamase (Bush and Bradford, 2019).

A high inhibitory potency of MEV was exhibited with serine carbapenemases, especially for KPC enzymes (Lapuebla et al., 2015; Castanheira et al., 2016). In addition, higher vaborbactam concentrations in MEV resulted in greater inhibition of isolates than meropenem alone (Castanheira et al., 2016). Similarly, based on *in-vitro* results, ICR was also reported as a promising potential agent for the treatment of carbapenemase-producing *Enterobacteriaceae* (Carpenter et al., 2019). Furthermore, ICR could prevent the resistance of *Pseudomonas aeruginosa* by inhibiting AmpC, while this effect was not reported in MEV (Lapuebla et al., 2015; Lapuebla et al., 2015). However, clinical studies investigating the clinical efficacy of carbapenem–β-lactamase inhibitor combinations remain limited. Therefore, this meta-analysis of all clinical trials comparing novel carbapenem–β-lactamase inhibitor combinations with other antibiotics was conducted to assess the efficacy and safety of ICR and MEV.

## Methods

### Search strategy

The current meta-analysis was based on reference to the Preferred Reporting Items for Systematic Reviews and Meta-Analyses (PRISMA) (Moher et al., 2009). Five databases, namely, PubMed, Cochrane Library, Embase, Ovid, and Web of Science, were searched at the end of 25 May 2021. Medical Subject Headings (“meropenem and vaborbactam” and “imipenem, cilastatin and relebactam”) and entry terms were used for literature retrieval.

### Selection criteria

Studies were included according to the following information: 1) study design: phase II or phase III trials or cohort studies; 2) interest: treatments contained at least MEV or ICR; 3) comparison or control: placebo or other equivalent drugs; 4) outcomes: clinical cure, microbial eradication, mortality, or adverse effects (AEs); and 5) language: written in English.

On the other hand, the exclusion criteria were based on the following aspects: 1) repetitive literature; 2) reviews, letters, or case reports; 3) animal or *in-vitro* studies; and 4) phase I trials or case–control studies.

### Data collection and risk of bias assessment

The potentially relevant articles were selected independently by two investigators (WY and PS) after deduplication by title and abstract. Any discrepancies were resolved through discussion with a third reviewer (YX). Several data details were extracted, including study registration number, first author, year of publication, diseases, treatments, study size, and outcomes.

Jadad score was used to assess the risk of bias in randomized controlled clinical trials, while the Newcastle-Ottawa Scale (NOS) was used in observational studies (Jadad et al., 1996; Margulis et al., 2014). The process of evaluation was completed by two independent authors (WY and YX).

### Statistical analysis

A random-effects model was used in our meta-analysis. Dichotomous data were used to assess the risk ratio (RR) for assessing the efficiency and safety of MEV or ICR. The heterogeneity was estimated with *I*-squared (*I* ^2^) statistic as follows: below 31% indicating low heterogeneity and little concern, 31% to 56% indicating medium heterogeneity, and greater than 56% indicating severe heterogeneity and considerable caution (Higgins and Thompson, 2002). Sensitivity analysis was used to evaluate the stability of the meta-analysis results. Subgroup analysis was performed according to different factors. A *P*-value less than 0.05 level was considered statistically significant. Publication bias was evaluated by Deeks’ funnel plot. All the above were calculated by Stata version 14 (StataCorp, College Station, TX, USA).

## Results

### Search results and quality assessment

**Figure 1:**
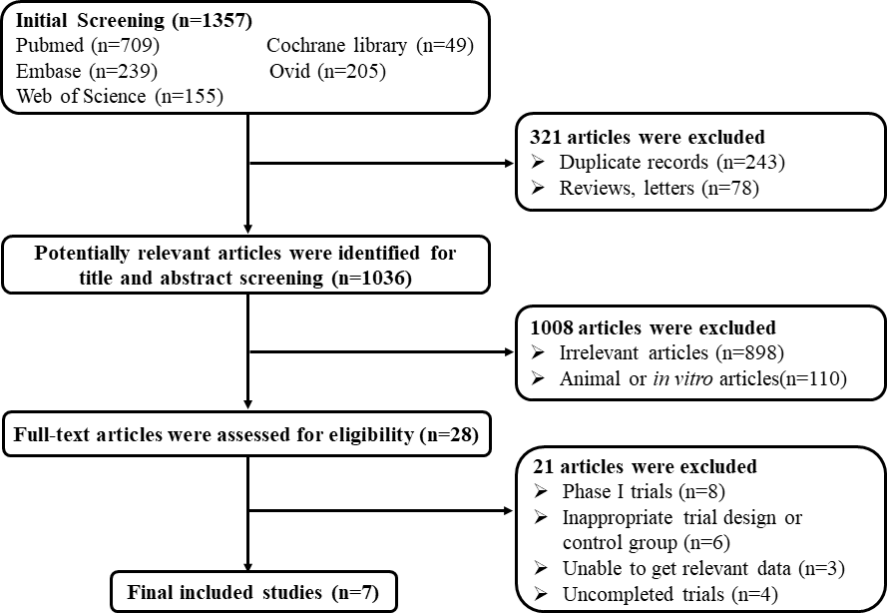
Flow diagram of the study selection.

According to the preset retrieval strategy, a total of 1,357 articles were retrieved. After deleting duplicates and reviews or letters, 1,036 articles were excluded by title and abstract screening. There were 28 full-text studies that were reviewed. Finally, seven articles were included in our systematic review (Lucasti et al., 2016; Sims et al., 2017; Kaye et al., 2018; Wunderink et al., 2018; Ackley et al., 2020; Motsch et al., 2020; Titov et al., 2020). The detailed selection process is presented in **Figure 1**.

In total, 1,984 patients were contained in this meta-analysis. There were six randomized controlled trials and one observational study. In addition, four studies evaluated the efficacy and safety of ICR, while three articles evaluated MEV (**Table 1**). All the included studies had high quality.

**Table 1:** Characteristics of the seven included studies.

Study registration no.	First author	Year of publication	Diseases	Therapy	Control	Study size	Bacteria	Ref.
NCT01506271	Lucasti C	2016	cIAIs	ICR	IMP+placebo	351	Gram-positive aerobic cocci Gram-negative aerobic bacilli	(Lucasti et al., 2016)
NCT01505634	Sims M	2017	cUTIs	ICR	IMP	302	Gram-positive aerobic cocci Gram-negative aerobic bacilli	(Sims et al., 2017)
NCT02452047	Motsch J	2020	Imipenem-non-susceptible bacterial infections	ICR	COL+ IMP	47	*Citrobacter freundii* *Enterobacter cloacae* *K. oxytoca* *K. pneumoniae* *P. aeruginosa*	(Motsch et al., 2020)
NCT02493764	Titov I	2020	HABP/VABP	ICR	PIP/TAZ	531	*K. pneumoniae* *P. aeruginosa* *Acinetobacter calcoaceticus-baumannii complex* *E. coli* *Methicillin-susceptible Staphylococcus aureus*	(Titov et al., 2020)
NCT02166476	Kaye KS	2018	cUTIs	MEV	PIP/TAZ	545	*E. coli* *K. pneumoniae* *Enterococcus faecalis* *Proteus mirabilis* *Enterobacter cloacae species complex*	(Kaye et al., 2018)
NCT02168946	Wunderink RG	2018	CRE infections	MEV	BAT	77	*E. coli* *K. pneumoniae* *Enterococcus cloacae* *Proteus mirabilis* *Serratia marcescens*	(Wunderink et al., 2018)
NA	Ackley R	2020	CRE infections	MEV	CZA	131	*E. coli* *K. pneumoniae* *Enterobacter cloacae* *Citrobacter*	(Ackley et al., 2020)

cIAIs, complicated intra-abdominal infections; cUTIs, complicated urinary tract infections; HABP/VABP, hospital-acquired/ventilator-associated bacterial pneumonia; CRE, carbapenem-resistant Enterobacteriaceae; ICR, imipenem–cilastatin/relebactam; MEV, meropenem–vaborbactam; IMP, imipenem–cilastatin; COL, colistin; PIP/TAZ, piperacillin/tazobactam; BAT, best available therapy; NA, not available; Ref., references.

### Clinical and microbiological response

**Figure 2:**
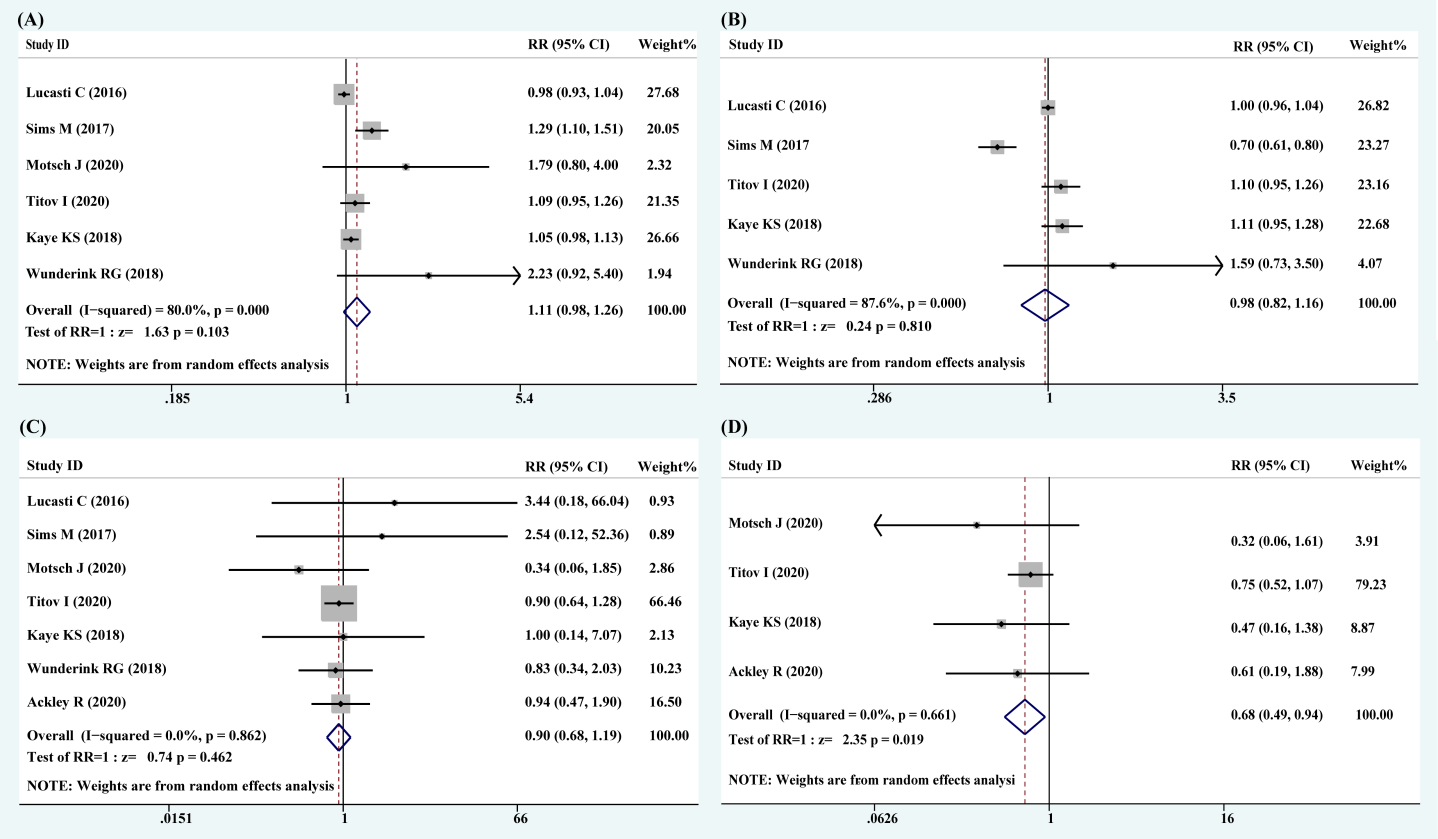
Forest plots of pooled clinical response, microbiological response, all-cause mortality, and 28-day mortality. **(A)** Clinical response; **(B)** microbiological response; **(C)** all-cause mortality; **(D)** 28-day mortality.

The most common pathogens identified at baseline were *Escherichia coli* (654 isolates), *K. pneumoniae* (377 isolates), and *P. aeruginosa* (109 isolates). Except for Wunderink’s and Ackley’s studies, the other studies have included a small number of Gram-positive bacteria. Six articles, including 1,437 patients, reported the rate of clinical response. The RR of clinical cure rate was 1.11 (95% CI: 0.98–1.26; *I* ^2^ = 80.0%, *P* = 0.103), indicating that clinical response among novel carbapenem–β-lactamase inhibitor combinations had a 1.11 times higher efficiency than that in comparators (**Figure 2A**).

The data related to microbiological response were analyzed from five studies with 1,307 patients. The RR of microbiological eradication rate was 0.98 (95% CI: 0.82–1.16; *I* ^2^ = 87.6%, *P* = 0.810), indicating that the microbiological response in the treatments with novel carbapenem–β-lactamase inhibitor combinations was 0.98 times of lower efficiency than that in comparators (**Figure 2B**).

**Figure 3:**
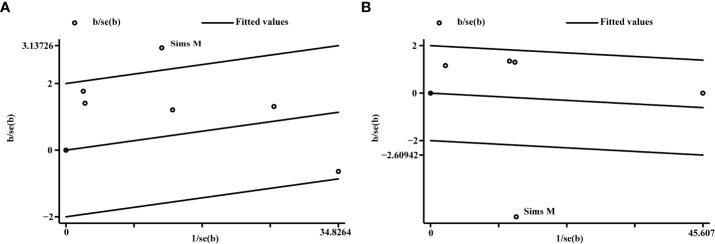
The Galbraith plot of clinical and microbiological responses. **(A)** Clinical response; **(B)** microbiological response.

It is of note that the pooled results of clinical and microbiological responses showed high heterogeneity. Furthermore, the Galbraith plot revealed that the study of Sims et al. was the main source of heterogeneity (**Figure 3**).

### Mortality and safety

There were seven articles that reported all-cause mortality and four articles that reported 28-day mortality. The RRs of all-cause mortality and 28-day mortality were 0.90 (95% CI: 0.49–0.94; *I* ^2^ = 0.0%, *P* = 0.862) and 0.68 (95% CI: 0.49–0.94; *I* ^2^ = 0.0%, *P* = 0.019), respectively (**Figures 2C, D**). A statistically significant improvement in survival rate was noticed with novel carbapenem–β-lactamase inhibitor combinations.

The therapy with novel carbapenem–β-lactamase inhibitor combinations resulted in similar AEs and serious adverse events (SAEs) compared to comparators (**Table 2**). The RRs of AEs from any cause, SAEs, and drug discontinuation due to drug-related AEs were 0.98 (95% CI: 0.93–1.04), 0.81 (95% CI: 0.65–1.01), and 0.66 (95% CI: 0.35–1.26), respectively.

**Table 2:** The risk of treatment-emergent adverse events.

AEs	RR	*I* ^2^	*P*-value
Any AEs	0.98 (0.93–1.04)	0.00%	0.548
Drug-related AEs	1.01 (0.75–1.36)	27.70%	0.923
SAEs	0.81 (0.65–1.01)	0.00%	0.058
Drug-related SAEs	0.64 (0.19–2.14)	0.00%	0.470
Drug discontinuation due to AEs	0.72 (0.45–1.16)	0.00%	0.177
Drug discontinuation due to drug-related AEs	0.66 (0.35–1.26)	11.30%	0.206

AEs, adverse events; SAEs, serious adverse events; RR, relative risk; I^2^, I-squared value.

### Sensitivity analysis

Sensitivity analysis was performed to estimate the influence of a single study on the overall results of the meta-analysis. The pooled results showed the change after deleting each trial (**Supplementary Figure 1**). After deleting each trial, the change of RR for clinical response was stable, indicating that the results were robust and reliable. However, for microbiological response, *I* ^2^ was changed after deleting the study of Sims et al., indicating that this study might be the most important cause of heterogeneity for microbiological eradication.

### Subgroup analysis

Subgroup analysis of clinical and microbiological responses was performed according to antibiotics, phase of the trials, and study size. As shown in **Table 3**, the clinical and microbiological response rates in patients receiving treatment with MEV (RR: 1.37, 95% CI: 0.62–3.01 and RR: 1.12, 95% CI: 0.97–1.30) were substantially higher than those receiving comparators. The rate of clinical response in phase II trials (RR: 1.12, 95% CI: 0.77–1.62) was similar to phase III trials (RR: 1.10, 95% CI: 0.96–1.27), while the rate of microbiological response in phase II trials (RR: 0.84, 95% CI: 0.48–1.44) was arithmetically inferior to phase III trials (RR: 1.10, 95% CI: 1.00–1.23). The difference in antibiotics and phase of the trials was not statistically significant (*P* > 0.05). It is of note that the number of patients less than 200 derived better clinical (RR: 1.97, 95% CI: 1.09–3.58, *P* = 0.026) and microbiological responses (RR: 1.59, 95% CI: 0.73–3.50, *P* = 0.245) in novel carbapenem–β-lactamase inhibitor combinations than comparators.

**Table 3:** Subgroup analyses of clinical and microbiological responses.

Subgroups		Clinical response				Microbiological response			
		RR	95% CI	I^2^	*P*-value	RR	95% CI	*I* ^2^	*P*-value
Antibiotics	ICR	1.14	(0.92–1.41)	86.6%	0.239	0.92	(0.73–1.15)	93.1%	0.457
	MEV	1.37	(0.62–3.01)	70.7%	0.431	1.12	(0.97–1.30)	0.0%	0.124
Phase of trials	II	1.12	(0.77–1.62)	94.8%	0.558	0.84	(0.48–1.44)	98.3%	0.520
	III	1.10	(0.96–1.27)	45.0%	0.171	1.10	(1.00–1.23)	0.0%	0.045
Study size	>200	1.08	(0.97–1.21)	81.6%	0.177	0.96	(0.81–1.14)	90.1%	0.638
	<200	1.97	(1.09–3.58)	0.0%	0.026	1.59	(0.73–3.50)	0.0%	0.245

ICR, imipenem–cilastatin/relebactam; MEV, meropenem–vaborbactam; RR, relative risk; 95% CI, 95% confidence intervals; I^2^, I-squared value.

### 3.6 Publication bias

The publication bias was assessed by funnel plot asymmetry. No potential publication bias was found in this study (**Supplementary Figure 2**).

## Discussion

Novel carbapenem–β-lactamase inhibitor combinations have provided promising therapeutic options against MDR organisms (Castanheira et al., 2016; Carpenter et al., 2019). To better understand the usefulness of ICR and MEV, this study reports the integrated analysis of seven studies (Lucasti et al., 2016; Sims et al., 2017; Kaye et al., 2018; Wunderink et al., 2018; Ackley et al., 2020; Motsch et al., 2020; Titov et al., 2020). The results demonstrated that novel carbapenem–β-lactamase inhibitor combinations had a higher clinical response rate and lower mortality than comparators, especially for MEV. In addition, both ICR and MEV were well-tolerated, including a significantly lower incidence of SAEs and study discontinuation.

In the integrated analysis of novel carbapenem–β-lactamase inhibitor combinations, treatment with novel carbapenem–β-lactamase inhibitor combinations was associated with high rates of favorable clinical response, while no obvious advantage of microbiological response was observed. Further subgroup analysis demonstrated that the rates of clinical cure and microbiological eradication using MEV were higher than the comparators (RR: 1.37, 95% CI: 0.62–3.01 and RR: 1.12, 95% CI: 0.97–1.30); however, ICR only showed a higher clinical response (RR: 1.14, 95% CI: 0.92–1.41). This is mainly because the effectiveness of MEV in the three included studies was mainly conducted in patients with *Enterobacteriaceae* (Kaye et al., 2018; Wunderink et al., 2018; Ackley et al., 2020). In addition, the results of the ICR led to conflicting conclusions for microbiological response. The Galbraith plot and sensitivity analysis revealed that the phase II trial of ICR against complicated urinary tract infections was the main source of significant heterogeneity for microbiological response (Sims et al., 2017). In this study, microbiological response rate exceeded 95% in a microbiologically evaluable population of patients of ICR and imipenem–cilastatin combined with placebo after the discontinuation of intravenous therapy visit. However, a decrease in microbiological response was observed at the early follow-up visit. One reason is that most of the included patients had imipenem-susceptible pathogens. Another reason is that the underlying anatomic or functional abnormalities could predispose these patients to recurrent infections (Sims et al., 2017).

The pooled data suggested that novel carbapenem–β-lactamase inhibitor combinations were associated with lower 28-day mortality. However, the all-cause mortality of ICR in two phase II trials was higher than that of imipenem–cilastatin (Lucasti et al., 2016; Sims et al., 2017). Two phase III trials demonstrated that mortality was lower with ICR than with the comparators (Motsch et al., 2020; Titov et al., 2020). Compared to ICR, the pooled data showed a consistently superior effect of MEV in terms of reduction of mortality (Kaye et al., 2018; Wunderink et al., 2018; Ackley et al., 2020). This may be related to the different pathogens among the included studies. The most frequent primary pathogens in the MEV studies were *Enterobacteriaceae* (Kaye et al., 2018; Wunderink et al., 2018; Ackley et al., 2020). Previous *in-vitro* models revealed that MEV had a higher barrier to resistance for KPC-producing *Enterobacteriaceae* than CZA (Sun et al., 2017; Sabet et al., 2018).

In the safety analysis, novel carbapenem–β-lactamase inhibitor combinations had a similar risk of AEs and drug-related adverse events to the comparators. However, the proportions of patients who experienced severe adverse events, severe drug-related adverse events, and drug discontinuation were lower than those given comparators. These are supported by the low heterogeneity identified when summarizing the effects across novel carbapenem–β-lactamase inhibitor combinations. It is of note that nephrotoxicity management is challenging in patients with serious Gram-negative infections (Lewis and Mueller, 2016; Pouch and Satlin, 2017). However, the treatment-emergent nephrotoxicity in ICR and MEV was lower than that in the comparators (Wunderink et al., 2018; Motsch et al., 2020). Hence, this intergrade analysis demonstrated that ICR and MEV appear to be safe and well-tolerated.

In recent years, special interest has arisen in developing carbapenem–β-lactamase inhibitor combinations. Except for ICR and MEV, other novel carbapenem–β-lactamase inhibitor combinations such as durlobactam, nacubactam, WCK-4234, taniborbactam, QPX7728, LN-1-255, and ANT2681 combined with imipenem or meropenem are in the development stage (**Supplementary Table 1**) (Vázquez-Ucha et al., 2020). Only sulbactam/durlobactam/imipinem–cilastatin and meropenem–nacubactam are undergoing clinical trials. It is of note that meropenem–ANT2681 exhibited activity against the metallo-β-lactamases producing CRE, while meropenem–QPX7728 showed activity against both serine-β-lactamases and metallo-β-lactamases *in vitro* (Everett et al., 2018; Lomovskaya et al., 2020). Future randomized controlled trials should assess the efficacy of these novel carbapenem–β-lactamase inhibitor combinations against carbapenem-resistant infections.

There are several limitations of the present work. First, only a small sample was included in this analysis. Second, the number of CRE was limited. Third, this integrated analysis lacks the detailed characterization of pathogenic bacteria, such as the types, subtypes, susceptibility to antibiotics, secretion of enzymes, and antibiotic resistance mechanisms. Moving forward, ongoing studies need to explore the optimal treatments with MEV and ICR.

## Conclusions

In conclusion, ICR and MEV were associated with an increase in clinical cure, microbiological eradication, and survival rate compared to comparators in phase III trials. In addition, the two novel carbapenem–β-lactamase inhibitor combinations are well-tolerated and could be applied as antimicrobial armamentarium against complicated infections.

## Data availability statement

The original contributions presented in the study are included in the article/**Supplementary Material**. Further inquiries can be directed to the corresponding author.

## Author contributions

The work presented here was carried out by all authors. WY and YX developed the concept and designed the study. WY and PS carried out the literature search and study selection. QL and LX co-worked on associated data collection. The quality of the included studies was checked by WY and YX. Disagreements were resolved by YX. Data synthesis and analysis were carried out by WY and PS. The manuscript was written by WY and corrected by YX. All authors discussed the results and implications and commented on the manuscript at all stages. All authors contributed to the article and approved the submitted version.

## Funding

This study was funded by the Key Research and Development Program of Zhejiang Province (No. 2021C03068).

## Conflict of interest

The authors declare that the research was conducted in the absence of any commercial or financial relationships that could be construed as a potential conflict of interest.

## Publisher’s note

All claims expressed in this article are solely those of the authors and do not necessarily represent those of their affiliated organizations, or those of the publisher, the editors and the reviewers. Any product that may be evaluated in this article, or claim that may be made by its manufacturer, is not guaranteed or endorsed by the publisher.

## Supplementary material

Supplementary Figure 1 Sensitivity analysis of clinical and microbiological response. (a) clinical response; (b) microbiological response.

Supplementary Figure 2 The Deeks’ funnel plot.
