# Supplementary material for: Efficacy and safety of novel carbapenem–β-lactamase inhibitor combinations: Results from phase II and III trials
Source: Front Cell Infect Microbiol. 2022 Sep 23;12:925662. doi: 10.3389/fcimb.2022.925662 (PMC9538188; doi:10.3389/fcimb.2022.925662)
Supplement: Supplementary file 1 [file Table_1.pdf]

Supplementary Table 1. Inhibition of major carbapenemases by novel carbapenem-β-lactamase inhibitor in the development process

β-lactamase inhibitors	Combination	KPC	NDM	VIM	IMP	OXA-23	OXA-48	Phase of Clinical Trial	ClinicalTrials.gov Identifier	Recruitment Status
Diazabicyclooctane-derived										
Durlobactam	Sulbactam/Imipinem-cilastatin	+	-	-	-	+	+	III	NCT03894046	Completed
Nacubactam	Meropenem	+	-	-	-	-	-	I	NCT03182504	Completed
WCK-4234	Meropenem	+	-	-	-	+	+		NA	
Boronic acid derived										
Taniborbactam	Meropenem	+	+	+	-	-	+		NA	
QPX7728	Meropenem	+	+	+	+	+	+		NA	
β-lactam-derived										
LN-1-255	Imipenem or Meropenem	-	-	-	-	+	+		NA	
Pyridine-2-carboxylic acid										
ANT2681	Meropenem	-	+	-	-	-	-		NA	

NA, not available.
