# Supplementary figures and images for: Efficacy and safety of novel carbapenem–β-lactamase inhibitor combinations: Results from phase II and III trials
Source: Front Cell Infect Microbiol. 2022 Sep 23;12:925662. doi: 10.3389/fcimb.2022.925662 (PMC9538188; doi:10.3389/fcimb.2022.925662)

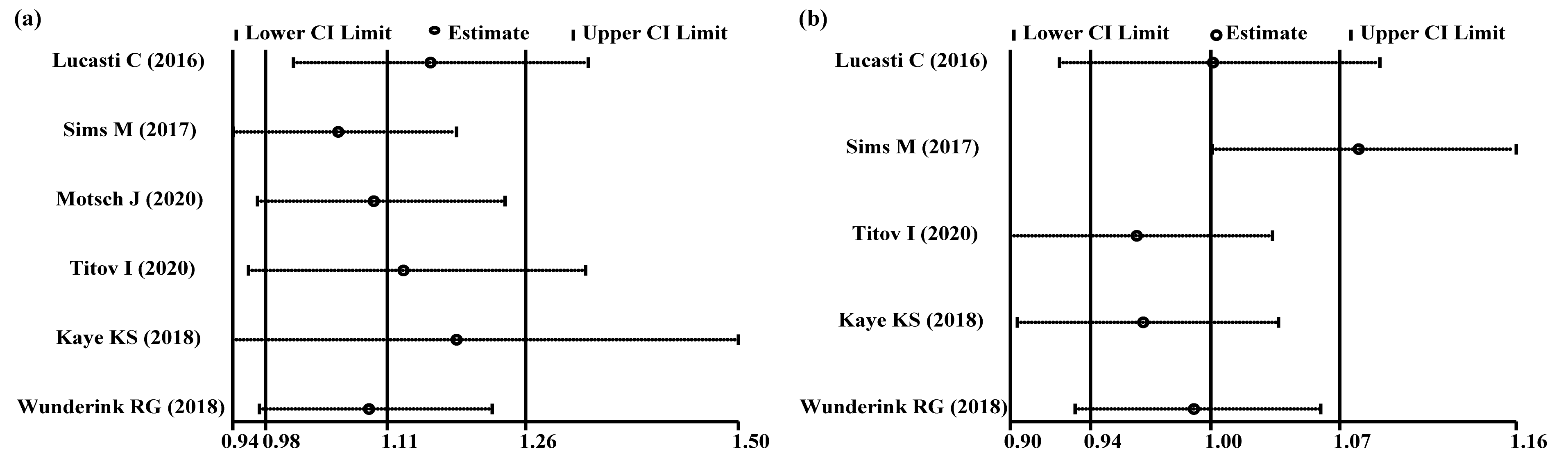

Supplement: Supplementary Figure 1 — Sensitivity analysis of clinical and microbiological response. (a) clinical response; (b) microbiological response. [file Image_1.tif]

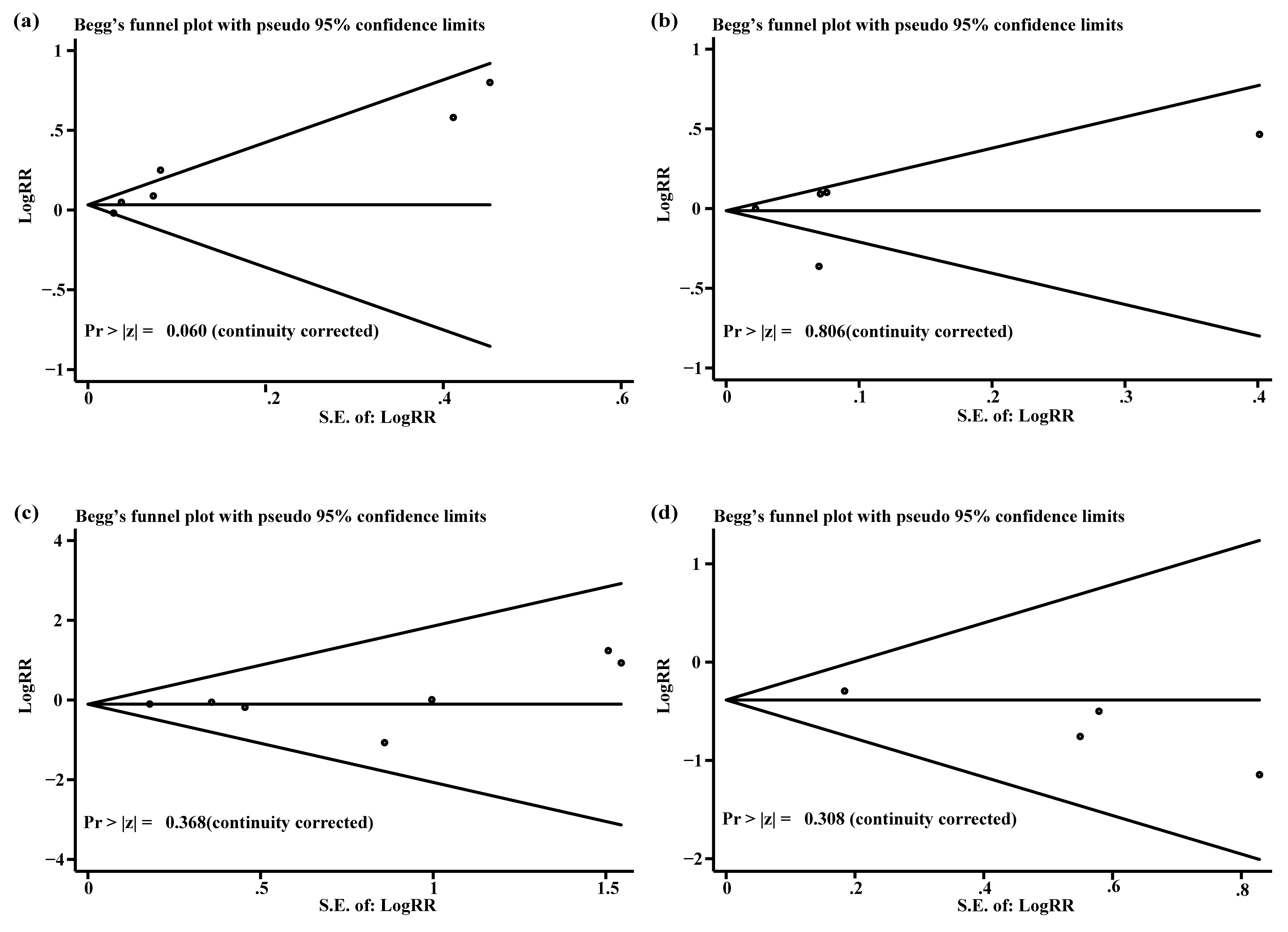

Supplement: Supplementary Figure 2 — The Deeks’ funnel plot. [file Image_2.tif]
